# Amyloid misfolding, aggregation, and the early onset of protein deposition diseases: insights from AFM experiments and computational analyses

**DOI:** 10.3934/molsci.2015.3.190

**Published:** 2015-05-17

**Authors:** Yuri L. Lyubchenko

**Affiliations:** Department of Pharmaceutical Sciences, University of Nebraska Medical Center, Omaha, NE 68198, USA

**Keywords:** nanomedicine, nanoimaging, atomic force microscopy, AFM, force spectroscopy, amyloids, Alzheimer’s disease, Parkinson’s disease, Huntington’s disease

## Abstract

The development of Alzheimer’s disease is believed to be caused by the assembly of amyloid β proteins into aggregates and the formation of extracellular senile plaques. Similar models suggest that structural misfolding and aggregation of proteins are associated with the early onset of diseases such as Parkinson’s, Huntington’s, and other protein deposition diseases. Initially, the aggregates were structurally characterized by traditional techniques such as x-ray crystallography, NMR, electron microscopy, and AFM. However, data regarding the structures formed during the early stages of the aggregation process were unknown. Experimental models of protein deposition diseases have demonstrated that the small oligomeric species have significant neurotoxicity. This highlights the urgent need to discover the properties of these species, to enable the development of efficient diagnostic and therapeutic strategies. The oligomers exist transiently, making it impossible to use traditional structural techniques to study their characteristics. The recent implementation of single-molecule imaging and probing techniques that are capable of probing transient states have enabled the properties of these oligomers to be characterized. Additionally, powerful computational techniques capable of structurally analyzing oligomers at the atomic level advanced our understanding of the amyloid aggregation problem. This review outlines the progress in AFM experimental studies and computational analyses with a primary focus on understanding the very first stage of the aggregation process. Experimental approaches can aid in the development of novel sensitive diagnostic and preventive strategies for protein deposition diseases, and several examples of these approaches will be discussed.

## 1. Introduction

Deregulation of the protein self-assembly process and the failure of cells to metabolize protein aggregates results in amyloid formation and is associated with a wide range of human diseases, termed *protein deposition (misfolding) disorders.* These disorders include *Alzheimer’s disease (AD)*, *Parkinson’s disease (PD)*, Huntington’s disease, systemic and localized amyloidosis, and transmissible encephalopathies [1]. The primary and perhaps most important causative factor in most neurodegenerative processes is the generation of aggregated proteins. Such proteins assemblies induce cellular stress and activate immunity in neurodegenerative diseases, resulting in neuronal dysfunction and cell death. The abnormal protein aggregates causes toxicity by disrupting intracellular transport, overwhelming protein degradation pathways, and/or disturbing vital cell functions. In addition, the formation of inclusion bodies is known to be a primary problem associated with the recombinant production of therapeutic proteins [2]. Formulation of these therapeutic proteins into delivery systems and their *in vivo* delivery are often complicated by protein association [3]. Additionally, since protein refolding is frequently accompanied by transient association of partially folded intermediates, the propensity to aggregate is considered a general characteristic of the majority of proteins and such aggregation-prone protein conformations are termed misfolded states [1,4,5]. Recent *in silico* analyses provided more specifics to the term “misfolding” that will be discussed below. Current demographic trends indicate the need for age-related and other degenerative disorder therapeutics, and macromolecular therapeutics will be at the forefront of future medical developments. Therefore, the field of medicine can be dramatically advanced by establishing a fundamental understanding of the key factors that lead to the self-aggregation of proteins involved in various protein folding pathologies.

Amyloid fibrillogenesis can be studied using a wide arsenal of modern biophysical approaches developed to analyze protein structures, conformational transitions, and folding processes [6]. Nanoimaging techniques such as transmission electron microscopy and atomic force microscopy (AFM) are excellent tools for distinguishing different morphologies of self-assembled aggregates. These techniques allowed several protein aggregate families to be classified: protofilaments (subfibrils), oligomers, and fibrils. An interesting feature of amyloid fibrils is their straight geometry [7], resulting in high stiffness and stability. These characteristics explain their resistance to proteolysis and their stable existence as brain plaques. A detailed analysis of the morphologies of different fibrils showed that the stiffness of amyloid fibrils varies broadly [8]. High stiffness is attributed to backbone hydrogen bonding and is modulated by side-chain interactions.

The use of high-resolution structural techniques, such as x-ray fibril diffraction and solid-state NMR, showed that misfolded proteins are assembled into fibrils with a periodic structure and are stabilized by β-sheet structural motifs [9]. Specifically, a structural model for amyloid fibrils formed by the 40-residue Aβ peptide Aβ (1–40) was proposed based on results of solid-state NMR spectroscopy [10,11]. These studies showed that the first ten residues of Aβ (1–40) are not structured, while the rest of the protein, except for the 25–29 region, adopts an β-sheet conformation. Residues 25–29 form the bend of the peptide backbone that brings the two β-strands in-register arrangement, creating a β-sheet (hairpin structure) through side chain-side chain interactions. Each Aβ (1–40) molecule in fibril assembled in stacks with a parallel arrangement of protein monomers. Crystallographic studies in the laboratory of D. Eisenberg [12] identified basic steric zipper structure as new fold, so some peptides are capable of forming different polymorphs with regard to their basic steric zipper structure, offering a possible explanation for amyloid polymorphism and prion strains [13].

On the basis of solid-state NMR and x-ray crystallography, the underlying structure of prion amyloids was found to share common structural features. A variety of different prion proteins, such as Ure2p1–89 (the basis of the [URE3] prion) [11,14], Ure2p10–39 [10,15], Sup35p ([PSI+] prion) [16,17], and Rnq1p ([PIN+] prion) [18,19], contain similarly arranged parallel β-sheets that are packed in-register. Additional studies led to a general model in which individual polypeptide chains are stacked in-register, forming parallel β sheets oriented perpendicular to the major axis of the fibril. However, later studies showed that this model, especially when it applies to proteins such as α-synuclein and yeast prions HET-s and Ure2p, should be replaced with a more complex one in which a part of the amyloid fibril polypeptide chain remains intrinsically disordered [20].

Although these studies provide useful information about the structure of large assemblies of amyloid proteins such as fibrils, the structural characterization of smaller assemblies such as oligomers remains unclear. Small oligomers, rather than larger assemblies, are neurotoxic and can induce the disorder. It is important to delineate the structure of smaller oligomeric assemblies to improve our understanding of their toxic characteristics [21,22,23]. Additionally, we need to know what conditions lead to the formation of these abnormal complexes, and more importantly, how to prevent their aggregation. Without a clear understanding of the protein misfolding and aggregation phenomena, there is little hope for the development of early diagnostic tools and efficient therapeutics capable of preventing the development of protein deposition diseases. Oligomers are transient species of the aggregation process that is composed of many steps and is accompanied by the formation of a variety of important intermediate states. Intermediate states are stabilized by weak interactions that are transient and difficult to measure [24], suggesting that non-traditional techniques have to be employed to probe these transient states.

Single-molecule biophysics techniques can help to probe molecular intermediates, follow molecular-scale events in real time, and measure a wide range of intermolecular interactions [25]. For example, conformational dynamics of the prion protein [26] and α-synuclein (α-Syn) [27] were studied with single-molecule fluorescence, including FRET. Use of the AFM pulling approach to study conformational transitions in α-Syn showed that disease-associated mutations in the protein facilitate its misfolding [28]. Interestingly, changes in environmental conditions, such as high ionic strength and the presence of Cu^2+^, which are known to increase the propensity of α-Syn to aggregate, shifted the conformational equilibrium toward β-like structures that are directly related to the aggregation of α-Syn [29]. Additionally, advances in high-power computational analyses made it possible to model the interaction of amyloid peptides with fibrils, enabling the characterization of the fibril and the dynamics of the amyloid fibril growth process at the atomic level [30,31]. This review describes recent advances in the application of AFM experimental methods and computational approaches to characterize amyloid dimers—the very first amyloid aggregate.

## 2. AFM force spectroscopy to probe intermolecular interactions

AFM instruments have dual capabilities [32]: imaging amyloid fibrils [33], and measuring the intermolecular interactions and mechanical properties of biological systems at the nanoscale level. Measuring intermolecular interactions is possible due to the ability to position the AFM probe with sub-nanometer accuracy in all three dimensions, and the ability to vary the stiffness of the AFM cantilever.

**Figure 1:**
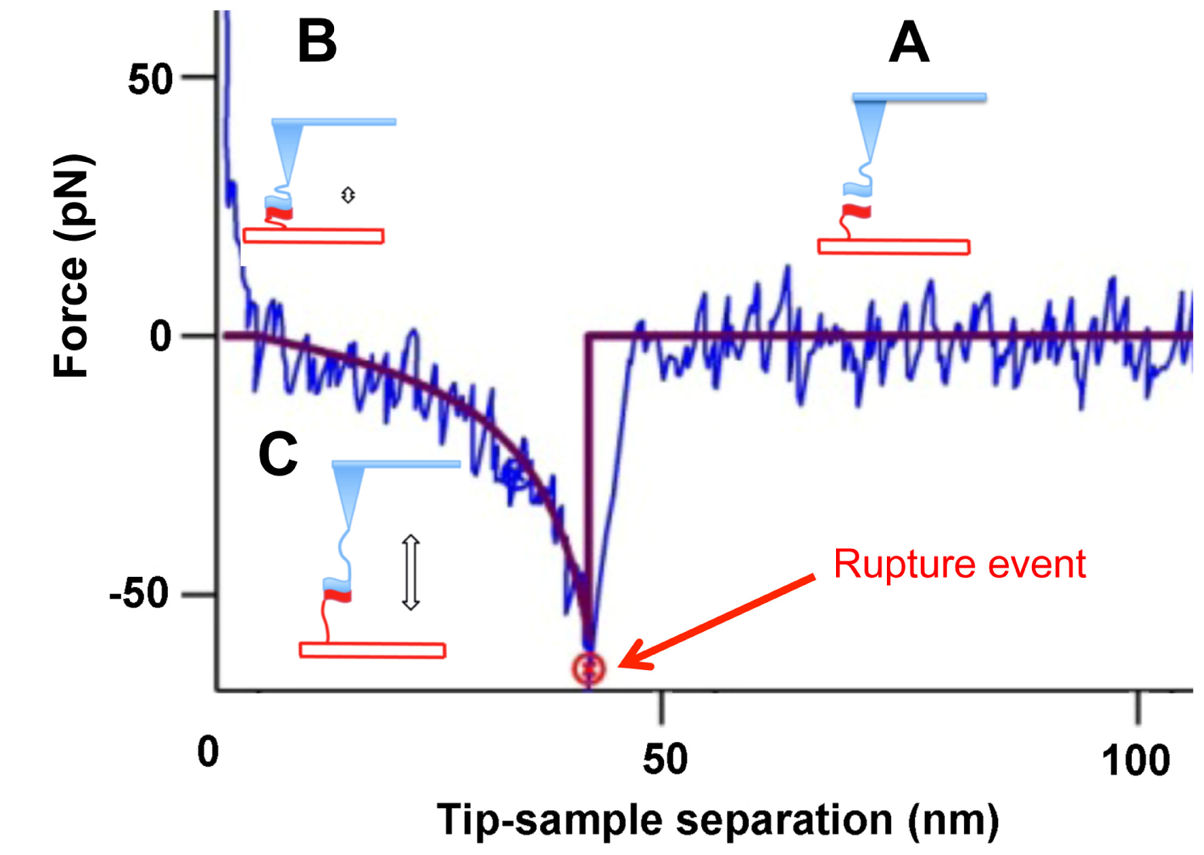
Schematic of AFM force spectroscopy experiment used to measure the interactions of molecules immobilized on the AFM tip and surface via flexible tethers The experimental force-distance curve is shown with blue. Red line is the fit of the experimental data with Worm-Like Chain (WLC) model. Cartoon (A) shows the approach stage at which tethered molecules are far from each other. Cartoon (B) illustrates the complex formation, which occurs at small tip-sample distance. The retraction of the tip as shown in cartoon (C) is accompanied by stretching of the tether and this entropy driven process is approximated with the WLC fit (red curve). After full stretching of the tether, the rupture occurs at the position indicated with a red arrow. After the complex rupture, there is no interaction between the molecules as evidenced by no change of the force on the tip-sample distance (horizontal red line).

AFM force spectroscopy is defined as the methodology of probing intermolecular and intramolecular interactions. In a typical force-probing experiment, the interactions between molecules that are immobilized onto the tip and the surface are probed, as schematically shown in Figure 1.

Experimental force-distance curve is shown with a blue line. Upon approaching the tip to the surface (Figure 1A), the molecules interact and form a complex (Figure 1B). Next, the tip is retracted, the tethers are stretched and this process is shown schematically in Figure 1C. Stretching of tethers is typically described by the Worm-Like Chain (WLC) model. The fit for this particular experiment is shown with a red line in the figure. When the pulling force is comparable with the strength of the interaction between the molecules, the complex ruptures. A sudden drop of the pulling force pointed by a red arrow in the figure is the signature of the rupture event.

The range of AFM spectroscopy applications is broad, varying from interactions of isolated molecules to interactions of a tip-immobilized ligand with receptors on a cell surface [34]. The feasibility of this approach to study complex biological systems has been proven by two back-to-back early publications that analyzed different systems. In one study [35], interactions between complementary DNA regions immobilized onto the AFM tip and the surface were probed. The DNA sequences were repeats of four nucleotides that enabled the formation of duplexes of different lengths. The AFM pulling approach was capable of detecting heterogeneity and characterizing the strength of the different duplexes. In another publication [36], biotin-avidin interactions were studied, enabling the structure of the biotin-avidin complex to be analyzed.

## 3. AFM probing of protein misfolding states

It was proposed in our early paper of McAllister et al. [37] that AFM force spectroscopy can be applied for probing transient misfolded states of amyloid proteins. The rationale for this stems from the fact that protein states facilitating the assembly in aggregates (misfolded states) differ from non-misfolded states by elevating protein-protein interaction forces. The hypothesis was tested by experiments in which the same protein was immobilized of the AFM tip and the substrate and the protein-protein interaction was measured as shown in Figure 1. The rupture force is low if the complex is weak (normal state of the protein), but the rupture force increases if the protein adopts the aggregation prone conformation. It is important to note that the described technique is entirely different from traditional approaches. It allows the unambiguous measurement of pair-wise protein-protein interactions, avoiding the effect of multiple interactions that complicate the quantitative analysis of the aggregation process. The approach was validated by comparing the force probing analysis with the amyloid aggregation analysis [7,37,38,39,40,41,42,43,44,45]. These studies showed that no interactions were observed during conditions in which aggregation did not occur. On the contrary, during conditions promoting aggregate formation, interprotein interactions were detected in a manner associated with the aggregation propensity of the protein. AFM spectroscopy was successfully used to probe amyloid protein dimers formed transiently during the AFM tip approach step.

**Figure 2:**
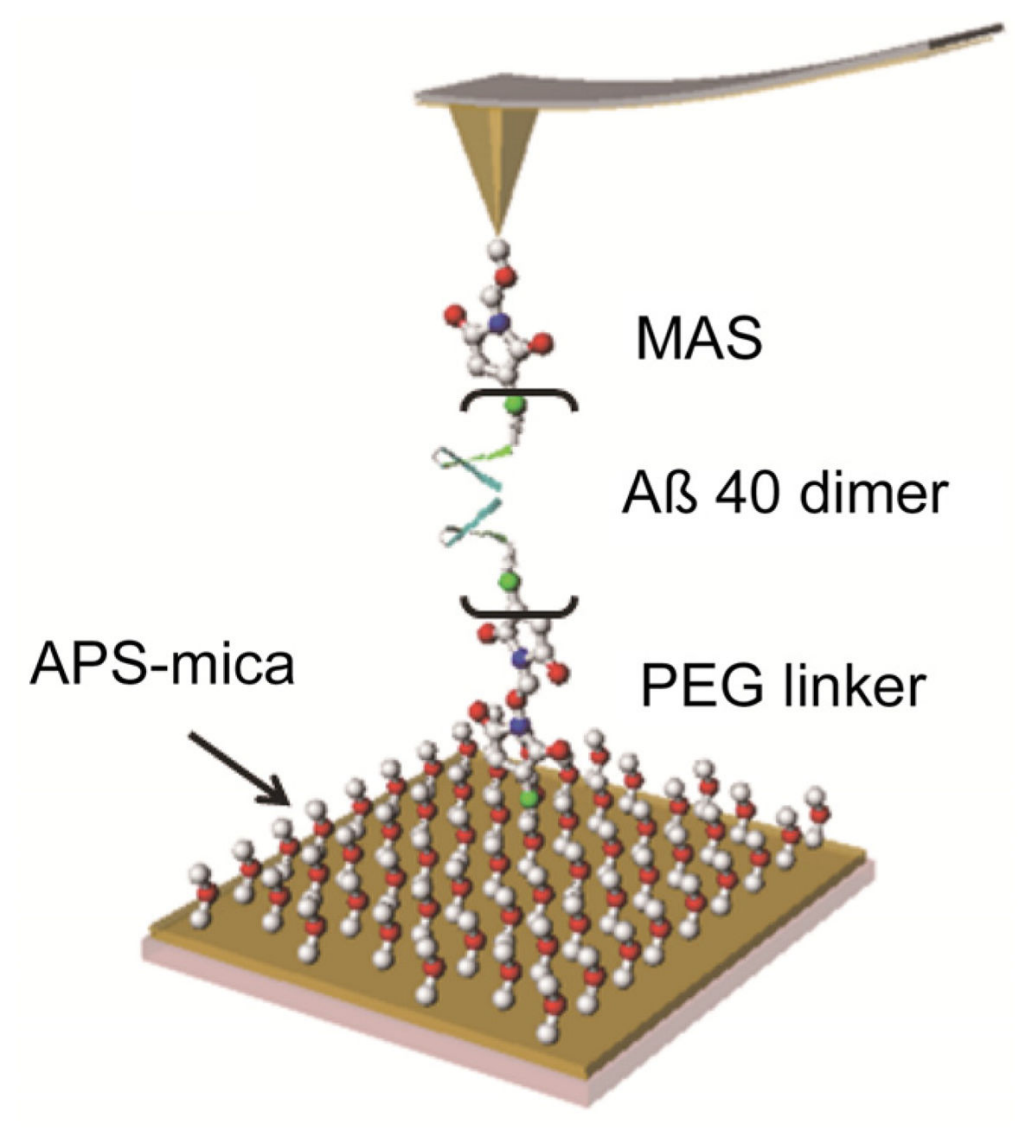
Schematic for single-molecule probing of interactions between amyloid β peptides Aβ 40 The protein is immobilized onto an APS functionalized mica surface via a flexible PEG tether. The same protein is also immobilized to the AFM tip using the MAS tethering method [38,42].

Figure 2 shows specifics to the single molecule AFM probing experiments that was developed to quantitatively characterize amyloid protein dimers formed transiently during the AFM tip approach step [7,37,38,42,43,44,45,46,47,48]. The Aβ40 proteins were covalently immobilized onto functionalized surfaces of AFM probe and mica substrates at their N-terminal cysteine residues with flexible tethers. The tethers play a dual role. First, they allow tethered protein to move freely around the anchoring point that allows the proteins to adopt the most optimal orientation for the dimer formation step. Second, prior to the dimer rupture, flexible tethers stretch; therefore, the length of the stretched tether as shown in Figure 1 defines the rupture process. Therefore, the position of the rupture event provides a fingerprint to the dimer rupture event that allows one to distinguish specific events from spurious ones corresponding to the non-specific interaction of the tip with the surface as it is illustrated in [49]. It was also demonstrated in that paper that the contour length measured after the WLC fit of force curves is sensitive to the tether length, therefore the contour length measurements can be applied to characterization of the protein folding within dimers. However, one should take into account the fact that the protein can be conjugated to any position on the tip surface. Probing by non-apically immobilized proteins is accompanied by shorter contour length values and difference depends on various factors as discussed in our paper [43]. For cooperatively dissociating systems such as short DNA duplexes the contour length distributions are quite narrow with the tether molecules heterogeneity being a rather substantial factor contributing to the width of the distribution [49]. However, the contribution of various non-apical locations to the contour length distribution depends on the density of the protein on the tip surface. At sparse coverage neighboring proteins can be too far from the one closest to the apical position, so such events will be rare. According to the analysis performed in our recent paper [43], such a sparse coverage can be achieved with the use of our surface chemistry and the protein immobilization methodology. The effect of the tip curvature on the contribution of the non-apical location of probes to the contour length distributions was analyzed recently in [17] and the simulated distributions are qualitatively similar to those obtained in our DNA pulling experiments [49].

Thus two major parameters of the AFM force spectroscopy experiments, the rupture force and the contour length can be used for the analysis of interaction of amyloid proteins as reviewed below.

## 4. Monomers folding within dimers

### 4.1. Amyloid β proteins

**Figure 3:**
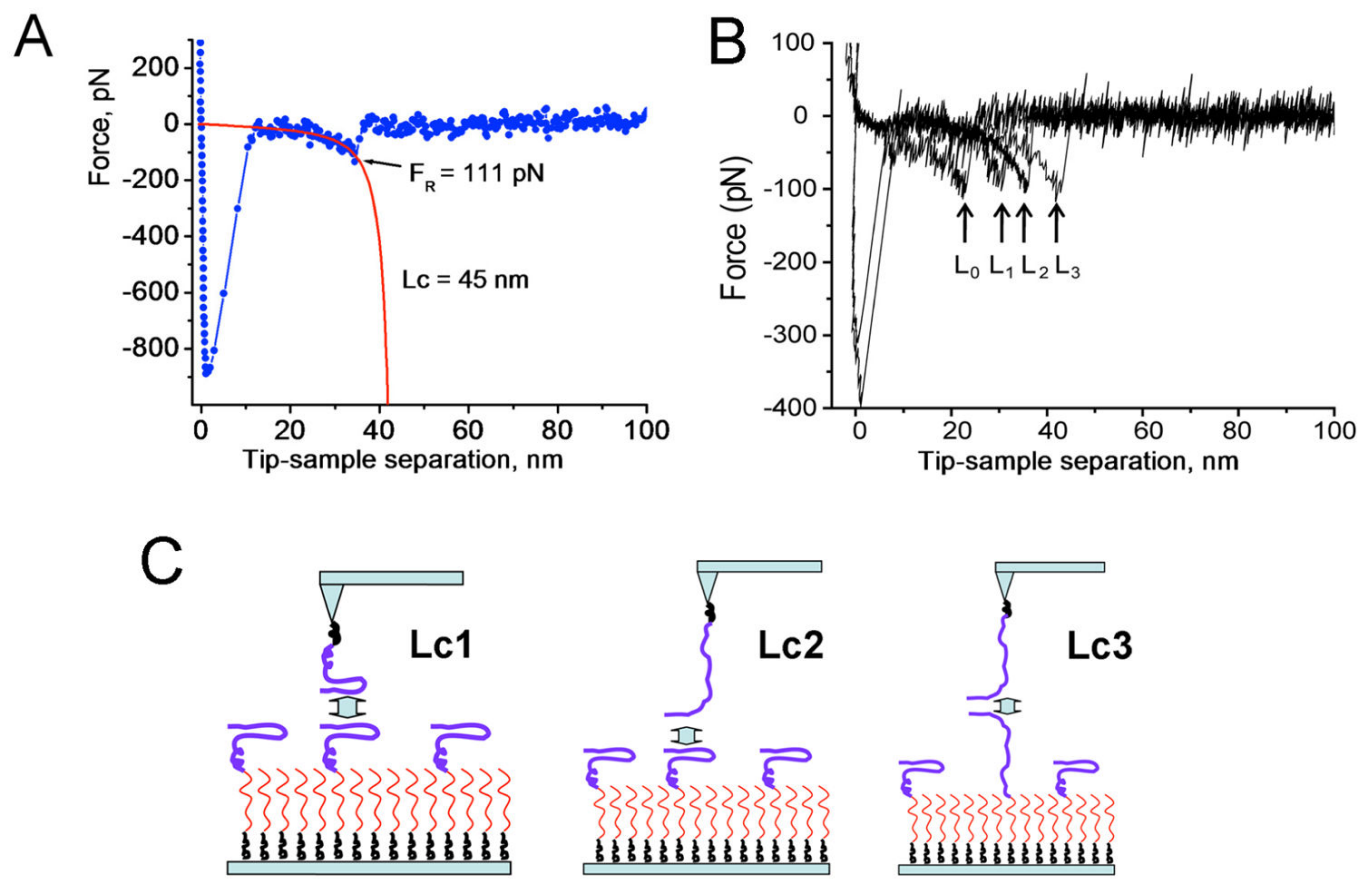
Contour length analysis of rupture events for Aβ 40 dimers (A) The individual force curve (blue) approximated with the worm-like chain model (WLC, red line). The values for the rupture force (F_R_ = 111 pN) and contour length (L_c_ = 45 nm) are indicated. (B) A set of four force curves with different rupture lengths. The rupture positions are indicated with arrows. (C) The model for the formation of misfolded Aβ40 dimers with different conformations corresponding to different contour lengths, L_c_1, L_c_2 and L_c_3, as measured in the force probing experiments. See paper [42] for details. Copyright © 2011, American Chemical Society.

Results in Figure 3 illustrate the application of single-molecule AFM spectroscopy to analyze interactions of amyloid β 40 (Aβ 40) protein [42]. Figure 3A shows the Aβ 40 dimer rupture event, with a red curve showing the approximation of the force curve with the worm-like chain (WLC) approach. This analysis enables the measurement of the contour length of a stretchable polymer that combines the length of the stretchable tether and the protein N-terminal segment involved in protein-protein contact within the dimer. Subtracting the tether length allows the position of the interacting segment to be defined. The experiments are typically repeated thousands of times, and statistical analysis of the results is performed. The analysis of the data set performed in [42] demonstrates that various contour lengths are present, as shown in Figure 3B. These data suggest that dimers with different conformations are formed. The rupture positions that correspond to different contour lengths are schematically shown in Figure 3C. The peptides can interact via β-hairpins, corresponding to the shorter contour length, L_c_1. The longer contour length, L_c2,_ represents the interaction between the hairpin and the C-terminal segment of the peptide. The longest contour length, L_c3_, corresponds to interactions via the C-terminal ends. The appearance of rupture events with a short contour length, L_c0_, suggests that N-terminal regions of the peptide are involved in peptide folding in the dimer, thereby shortening the non-structural N-terminal segment. This assumption is in agreement with the molecular dynamic simulation analysis [50].

The approach described above was used in [45] to characterize the difference in folding between Aβ 40 and Aβ 42 proteins. Aβ 42 is the same as Aβ 40 with the addition of two extra residues at the C-terminus.

**Figure 4:**
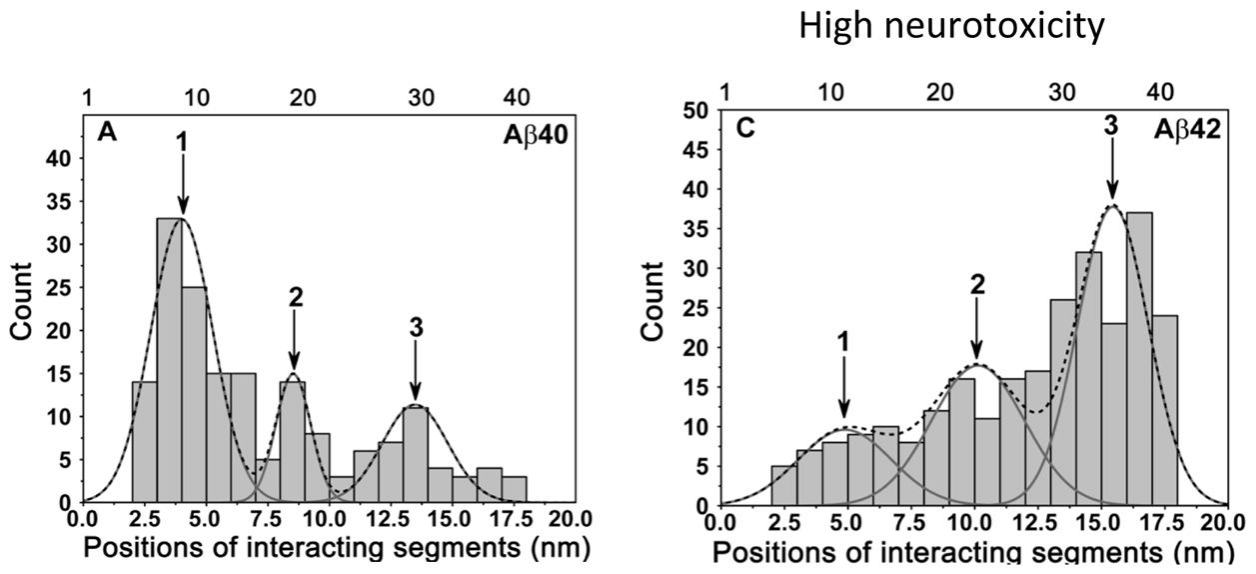
The contour length distributions for Aβ 40 and Aβ 42 dimers are shown in A and B, respectively The positions of interacting segments are aligned according to the contour length distribution. The dotted lines denote the overall distribution profiles and the peaks are approximated according to the profiles. The arrows indicate major interacting segments.

Figure 4 summarizes the results of the comparative force spectroscopy analysis and shows the contour length distributions for both proteins. The length distribution of the Aβ 40 N-terminal segments is shown in Figure 4A. The overall distribution is broad and was fit by three peaks. The width of the peaks was consistent with the contour length distributions for flexible polymers obtained in [49]. On this distribution, the predominant peak 1 corresponds to a segment of 1 ~ 10 residues, peak 2 corresponds to the longer segment of ~ 20 residues, and the third peak comprises almost 35 residues of the peptide from the N-terminus. This distribution suggests that Aβ 40 can adopt at least three primary conformations within the dimers that differ by the overall size of the peptide segments involved in the interpeptide interactions. The predominant conformation defined by peak 1 corresponds to a relatively short N-terminal segment peptide conformation. The remaining portion of the peptide is involved with dimer formation. Notably a very similar profile of contour lengths was obtained in [42] in which early studies of Aβ 40 dimers were studied.

A similar analysis was applied to the Aβ 42 peptide (Figure 4B). The comparison of Figs. 4A and B demonstrates that the extra two amino acids present in Aβ 42 dramatically alter its interaction pattern. The primary interactions that occur via the N-termini for Aβ 40 are replaced in Aβ 42 by interactions within the C-terminus (Peak 3, Figure 4B). This is consistent with a recent paper by Gu et al. [51], in which Aβ 42 oligomers were found to be tightly packed at the C-terminal region. The model on the effect of the N-terminus on the aggregation propensity of Aβ peptides is in agreement with the results of a recent paper [52] in which the interaction of Aβ 40 and Aβ 42 monomers in solution was studied. They found that Aβ 40 monomers form a type of antiparallel β-hairpin involving the central hydrophobic cluster (residues 16–21) with the N-terminal residues 9–13. The Aβ 40 monomers also form a major antiparallel β-hairpin involving the central hydrophobic cluster with the C-terminus. The contribution of the N-terminal residues to peptide interactions was also supported by Maji et al. [53]. They substituted Tyr throughout the peptide and found that Tyr substitution at Asp1 in both Aβ 40 and Aβ 42 leads to the slowest aggregation kinetics. These combined results reveal dramatic differences in the interaction patterns of Aβ 42 and Aβ 40 monomers within dimers. Although the sequence difference between the two peptides is at the C-termini, the N-terminal segment plays a key role in peptide interactions, modulating higher toxicity of Aβ 42.

### 4.2. Interactions of α Synuclein: Effect of familial mutations

**Figure 5:**
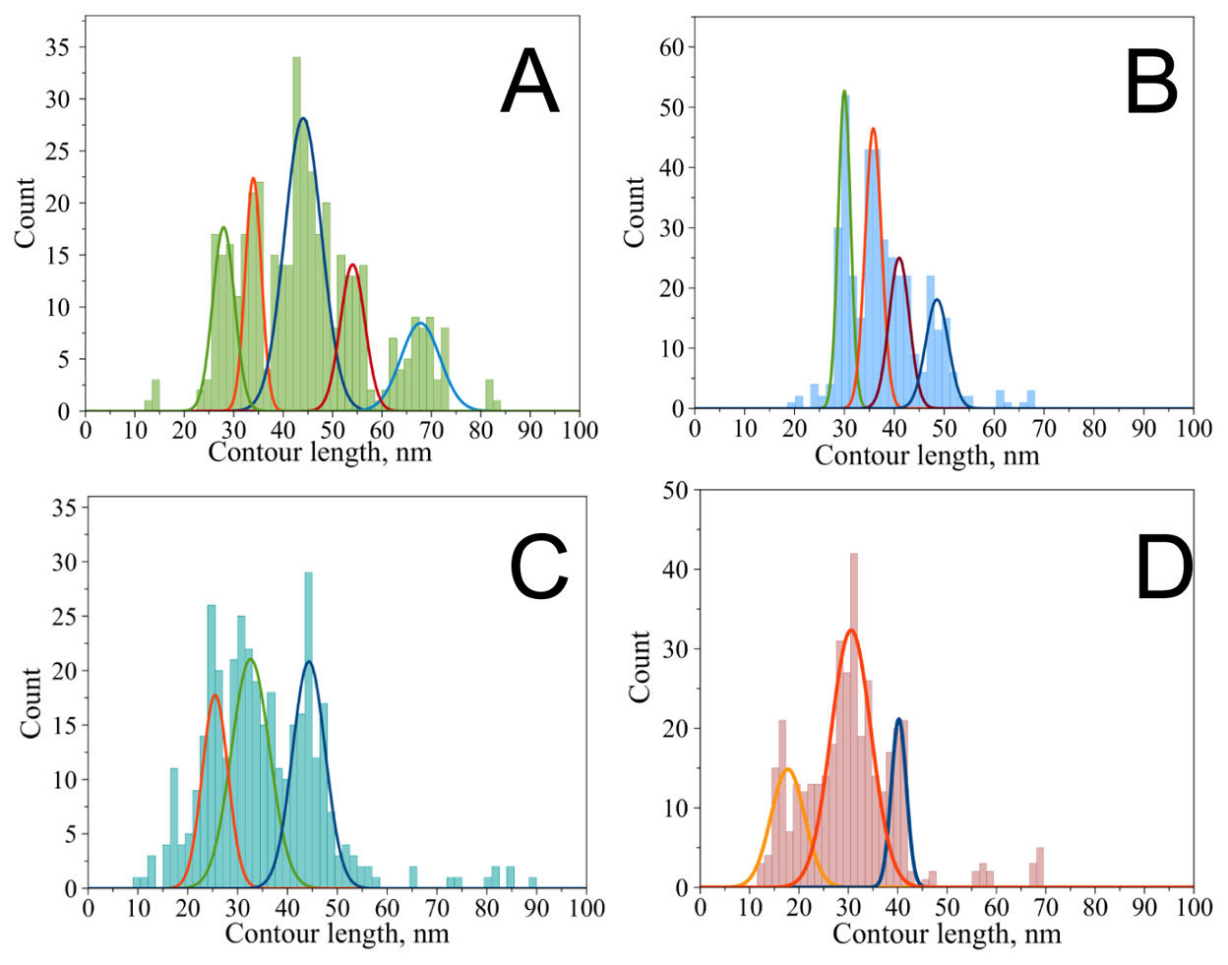
Contour length distribution for α-Syn variants—group B force-distance curves with a single rupture A) wild type [with maxima at 28 ± 2 nm (peak 0), 34 ± 2 nm (peak 1), 44 ± 4 nm (peak 2), 54 ± 3 nm (peak 3) and 68 ± 4 nm (peak 4)], B) A30P mutant (with maxima at 30 ± 1, 36 ± 2, 41 ± 2 and 49 ± 2 nm), C) A53T mutant (with maxima at 26 ± 3, 33 ± 4 and 44 ± 3 nm), D) E46K mutant (with maxima at 17 ± 3, 31 ± 4 and 40 ± 2 nm). The figure was reproduced from [54]. Copyright © 2013, American Chemical Society.

A similar approach was used to characterize the dimer assembly and protein aggregation processes of α-synuclein (α-Syn), which define the development of PD [54]. A statistical histogram of contour length (L_C_) values for WT α-Syn is shown in Figure 5A. The distribution has a set of clear features and was approximated with five peaks representing the most reproducible positions of the rupture events. A Gaussian fit of the histogram resulted in distribution maxima at 28 nm (*peak 0*), 34 nm (*peak 1*), 44 nm (*peak 2*), 54 nm (*peak 3*) and 68 nm (*peak 4*). The most prominent peak appears at 44 nm, which contains 42% of all detected single rupture curves for WT α-Syn. The fact that the distribution reveals several peaks suggests that there is more than one conformation of WT α-Syn capable of forming the dimer. The effect of three major familial PD mutations on α-Syn dimer formation was analyzed using the force spectroscopy approach.

Results of force-distance curve analysis for the α-Syn variants are shown in Figure 5B–D. Similar to the data for WT α-Syn, the distributions of the contour lengths for the α-Syn variants have specific features. The variant distributions appear simpler with fewer peaks that are slightly narrower than for those for WT α-Syn (Figure 5A–D). The distributions reveal different patterns for each α-Syn variant, suggesting the existence of more than one conformation within the dimer. The contour length measurements reveal that the contour length profiles are sensitive to the introduction of mutations. This can be observed without any additional analysis (Figure 5). The effect of the mutations is reflected by the change in interprotein interactions. We fitted the overall contour length distribution profiles with a set of peaks to facilitate the data interpretation. Four prominent peaks are clearly seen for the A30P mutant with the Gaussian maxima at 30, 36, 41 and 49 nm (Figure 5B). The A53T and E46K mutants had their contour length distributions skewed to shorter L_C_ values when compared to the distributions for WT and A30P (Figure 5C–D). The majority of the rupture events for all α-Syn variants appear within the range of ~ 26 nm to ~ 50 nm. Although there were other interactions observed at longer and shorter distances, these had a lower probability than the peaks in the 26–50 nm range. Moreover, other peaks outside of this region are not present for all the variants. For example, the shortest peak observed for E46K at 17 nm is not present in the histogram for the other variants. Additionally, the longest peak at 68 nm for WT α-Syn does not have a counterpart in the histograms for the mutants, suggesting that the interaction producing this long rupture event is specific for WT α-Syn.

Overall, single-molecule force spectroscopy performed in [54] showed that only a limited span of the C-terminal region of wild-type α-Syn is involved in the stabilization of dimers. The interactions correspond to a very short non-interacting segment ~ 122–140 aa which is further in the C-terminal region than expected based on NMR studies of fibrillar structures [55,56]. The pathological mutants, especially A53T and E46K, in the dimer have a higher propensity for β structure in the C-terminal part. This assumption is in agreement with ssNMR analysis of fibrils formed by A53T mutant that detected the formation of an extended β-sheet core in the C-terminal region as compared to WT α-Syn [55]. Interestingly, the single-point mutations located within the N-terminal region of the protein dramatically change the pattern of protein misfolding by extending interactions into the C-terminal region. These results suggest that the single point mutations affect distantly located interacting segments of the misfolded α-Syn. A30P, E46K, and A53T mutations directly cause the interacting regions located in close proximity to be destabilized. Importantly, compared to WT α-Syn, E46K and A53T mutations increase the dimerization propensity by promoting the simultaneous interactions of multiple segments, while the A30P mutation promotes single-type interactions. We hypothesized that these differences in interacting patterns define a protein’s aggregation pathway; however, future studies are needed to clarify this hypothesis.

### 4.3. Role cations on interactions of amyloid β proteins

**Figure 6:**
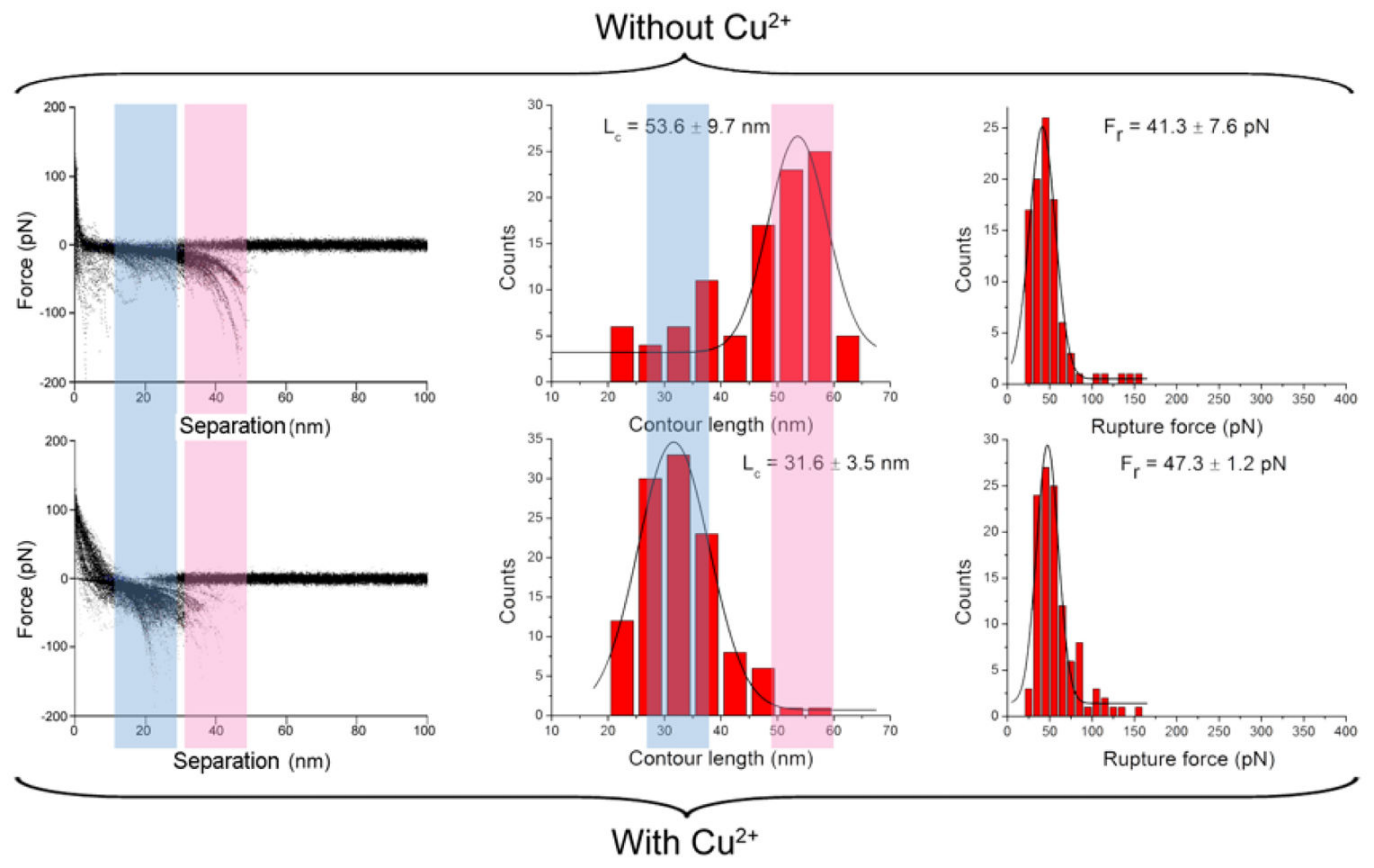
AFM spectroscopy in the presence and absence of Cu^2+^ cations at pH 7.4 The columns include, from left to right: the overlap of all raw force curves, the distribution of contour length (middle), and the distribution of rupture forces (right). The L_c_ and F_r_ denote the most probable contour length and the most probable rupture force, respectively. Figure was reproduced from [44]. Copyright © 2012, Springer Science+Business Media New York.

An unexpected finding was made in studies of the effect of Cu^2+^ cations on interactions of Aβ 42 monomers [44]. Given the fact that this divalent cation facilitates aggregation of Aβ 42 protein, it would be expected that the presence of Cu^2+^ cations would also increase interprotein interactions. Results shown in Figure 6 do not support this expectation. The rupture forces measured in the absence and presence of Cu^2+^ cations were essentially identical, whereas the contour length distributions were different for both experimental conditions. This finding demonstrates that Cu^2+^ cations change the protein folding patterns within dimers. Interestingly, in the presence of divalent cations the interaction is shifted toward the N-termini of the Aβ 42 monomers, suggesting that interactions between N-terminal segments are increased by Cu^2+^ cations. This finding is in agreement with Cu^2+^ cations binding to N-terminal histidines, and a recent study demonstrating that the N-terminus of Aβ 42 participates in the formation of a β-sheet conformation [57].

A similar approach was used to analyze the effect of cations on α-Syn [58]. The results demonstrate that Zn^2+^ or Al^3+^ cations dramatically increase interactions between α-Syn monomers, resulting in dimer formation at conditions that are unfavorable for α-Syn dimerization in the absence of the cations at neutral pH. Data also shows that Al^3+^ cations are more effective than Zn^2+^ cations at promoting monomeric interactions. The analysis of the contour lengths of the dimers rupture suggests that a part of the C-terminal segment of α-Syn is involved in monomeric interactions in the presence of the cations. The dimers assembly in the absence of the cations is different, leading to the conclusion that cations change the conformations of misfolded α-Syn. These and previous studies illustrate that AFM force spectroscopy is a nanotool capable of characterizing misfolded amyloid proteins and elucidating the role of environmental conditions in the disease-prone aggregation process.

### 4.4. Effect of spermidine on interactions of α Synuclein monomers

Cellular polyamines, such as spermidine, have multiple essential functions in living organisms, and their expression levels are maintained by highly regulated pathways. Altered metabolism may result in enhanced levels of polyamines. Recently, the AFM probing approach was applied to study the effect of intracellular metabolites, such as spermidine, on α-Syn misfolding [59]. It was found that the very first steps of α-Syn self-assembly are facilitated by the presence of the cellular polyamine spermidine. Importantly, the pathogenic mutation A30P does not increase the propensity of α-Syn to misfold in the presence of spermidine, but rather increases the strength of interprotein interactions and stabilizes the dimer structure. It was proposed that the mutation affects conformational preferences of monomeric α-Syn distinct from WT, as supported by several previous studies [60,61,62]. According to our data, the preferred conformations of the A30P mutant result in higher forces of intermolecular interactions, which might lead to a more rapid association of α-Syn molecules. The differences in the misfolding patterns between WT and A30P α-Syn might be responsible for the higher propensity of the mutant to aggregate and cause early-onset PD. The AFM data suggest that the presence of physiological concentrations of spermidine induces conformational changes in α-Syn, resulting in misfolded α-Syn monomers. Therefore, in addition to their normal cellular functions, biogenic polyamines may also have a pathogenic role in initializing the aggregation of α-Syn. Single-molecule force spectroscopy demonstrates that misfolded states of α-Syn formed in the presence of spermidine are more prone to undergo self-assembly than the normal state of the molecule. Importantly, more than one segment within the protein molecule might be responsible for the initial association of α-Syn into dimers, and possibly into higher-order oligomers and fibrils. This finding suggests that even the first step of α-Syn self-assembly (dimerization) possesses some degree of heterogeneity. It is hypothesized that these different misfolded conformations can lead to different types of oligomers and define the aggregation pathway.

The hypothesis in the spermidine role on the early onset of PD proposed in [59] was discussed in [63] in which the role of spermidine as a biomarker for PD progression was identified. The authors found elevated concentration of N8-acetyl spermidine in a fast motor progression disease phenotype suggesting that altered polyamine metabolism may be a predictive marker of rapidly progressing PD.

## 5. Molecular mechanisms of protein assembly into disease-prone aggregates

**Figure 7:**
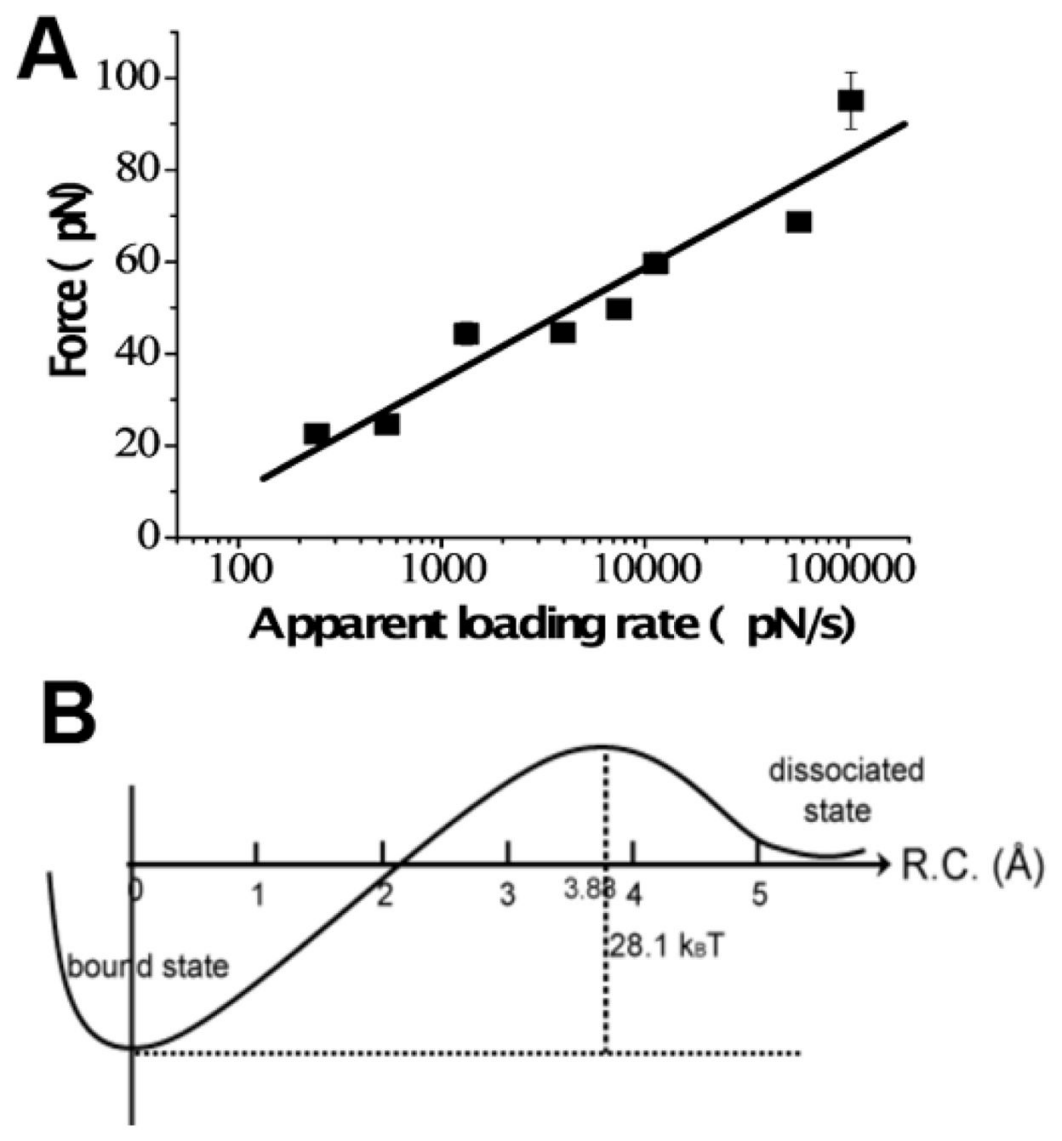
DFS analysis of α-Syn interactions (A) DFS plot for AFM probing of α-Syn interactions measured at pH 5.1. The linear relationship indicates that the dissociation of an α-Syn dimer follows a one-barrier path shown in (B). The energy of the barrier is 28.1 k_B_T; the complex lifetime is 0.27 ± 0.13 s. The figure was reproduced from [47]. Copyright © 2008, Elsevier Ltd..

The ability to perform force spectroscopy analysis at the single-molecule level [48] enables the application of dynamic force spectroscopy (DFS), a quantitative approach introduced by Evans and Ritchie [64]. In the DFS approach, pair-wise interactions are probed over a broad range of pulling rates. Rupture forces are determined for each force-distance curve, and force distributions are obtained and analyzed as described [48]. The results of DFS analysis for α-Syn are shown in Figure 7. The plot of the most probable force versus the logarithm of the pulling rate is shown in Figure 7A. The experimental data points are approximated with a linear plot, which according to the DFS theory suggests that the dimer dissociation is characterized by a one-barrier energy profile (Figure 7B), and the barrier height is 28 k_B_T, corresponding to an off-rate (dissociation rate) constant value of ~ 1 sec [47]. This suggests that dimers of α-Syn protein are stable and dissociate in the range of seconds. These measurements are in line with our recent results obtained by entirely different experimental approach—tethered single molecule fluorescence [65]. The dimer lifetime in that experiments was obtained directly from measurements of the fluorescence duration time produced by fluorescently labeled α-Syn monomer bound to the surface tethered α-Syn. These studies revealed two types of α-Syn dimers with lifetimes 197 ± 3 ms and 3334 ± 145 ms with the latter population comprising ~ 10% of the events.

Similar studies were performed on Aβ proteins of various sizes [42,66,67] and Sup35 peptides [68]. The common result of these studies is that dimers are highly stable, with lifetimes in the second timescale. This differs markedly from the dynamics of monomers, which occur on a microsecond-nanosecond timescale. These findings led to the hypothesis that the dimerization of amyloid-type proteins is a mechanism by which the aggregation-prone transient misfolded state of a protein is stabilized. A major conclusion of these studies is that the formation of dimers is the key step in the amyloid assembly process, and that the long lifespans of dimeric complexes promote the selection of misfolding-aggregation pathways rather than non-pathologic pathways [48]. However, other important questions, such as how the misfolded dimers form, cannot be answered by these experiments. They could be assembled from the transiently formed misfolded monomers; however, this assumption is not supported by experimental and computational studies that revealed that the structures of monomers did not resemble their conformations in fibrils. The structure of dimers could be different from the structure of fibrils, and recent computational results reviewed below address this important issue.

Initially, the issue of dimer assembly was analyzed in [67], in which all-atom Molecular Dynamics (MD) simulation was used to directly analyze the dimerization process of Aβ (14–23) peptide terminated with cysteine at its N-terminus [69]. This segment contains the “central hydrophobic cluster” of Aβ and is critically involved in Aβ aggregation [70].

**Figure 8:**
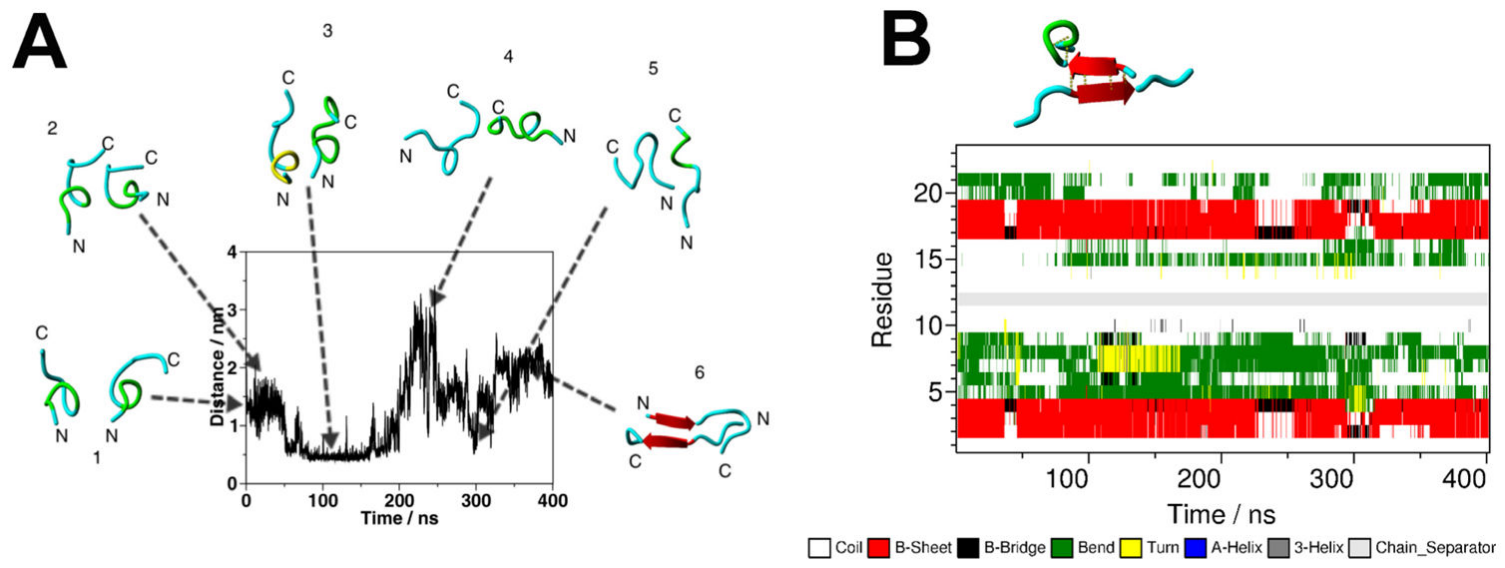
Computational studies of Aβ (14–23) interactions (A) The time-dependent of the N-N distance for the monomers, obtained from the all-atom MD simulation of two closely positioned monomers. Snapshots of the monomers conformations for selected points on the time trajectory are indicated above the graph. (B) Time-dependent secondary structure of the monomers (DSSP diagram) for the next 400 ns simulations. The snapshot for the representative conformation of the dimer is indicated above the graph. Amber-ff99sb*-ILDN force field was used in the simulations. The figure was reproduced from [67]. Copyright © 2013, American Chemical Society.

MD simulations of the dynamics of monomeric Aβ (14–23) peptide in an aqueous environment showed no formation of any extended β-sheet conformation. However, changes were observed when two monomers were brought into close proximity, allowing their interaction (Figure 8A). During the initial 350 ns period, the distance fluctuated between the monomers with no considerable change in the monomers secondary structure. After 350 ns, a rearrangement occurred that resulted in the formation of an antiparallel β-sheet conformation (red arrows in snapshot 6 in Figure A). Thus, the intermolecular interactions of the monomers triggered conformational changes within the individual peptide chain, which led to the formation of the antiparallel β-sheet structure. The arrangement of monomers in an antiparallel orientation leads to the cooperative formation of a β-sheet conformer. Moreover, the dimer remains stable during the next 400 ns simulation period, as shown in the DSSP diagram in Figure 8B.

The largest cluster of the trajectory contained 82.1% of the explored structures. A representative structure of the largest cluster is shown above the diagram. The antiparallel β-sheet structure of the dimer is stabilized not only by hydrogen bonds, but also by salt bridges and weakly polar interactions of the side chains. However, on the basis of data from solid-state NMR studies of fibrils assembled by Aβ (16–22) peptide, an in-register pairing of monomers in an antiparallel orientation is expected [71]. MD simulations confirmed these expectations, revealing dimers with an in-register assembly of Aβ (14–23) monomers in an antiparallel orientation [67]. These observations prompt the question: what Aβ (14–23) dimer structure is observed in AFM force spectroscopy experiments? This question was answered in the studies reported in paper [19], in which a novel computational approach was used to analyze AFM spectroscopy results. The direct comparison of experimental data with the computational results led to the conclusion that the out-of-register conformation of Aβ (14–23) dimers, shown in Figure 8B, is the predominant structure. However, the dimer structure is very dynamic, resulting in the formation of the in-register dimer conformation.

Misfolding is a rather old term coined to differentiate the structure of the protein in amyloid aggregates from the one relevant to its physiologic function. For example, lysozyme, which is enzymatically active in the well-defined tertiary structure obtained in crystallographic studies, for assembly in fibrils, unfolds and reassembles [72]. However, such proteins as Aβ proteins and α-Syn do not have well-defined secondary and tertiary structures and belong to the category of intrinsically disordered proteins [73]. At the same time, both proteins in fibrils are structured; therefore, misfolding term for such proteins means rearranging them in conformations that facilitate the self-assembly process. Here we use term “misfolding states” for proteins conformations that are characterized by elevated interprotein interactions; however, such misfolded states can be different form the protein conformation in amyloid fibrils. Indeed, our *in silico* analyses showed that monomers conformations within Aβ (14–23) dimers are different form the peptide conformation within fibrils [19,67]. Although β-sheet conformation presents in both assemblies of the peptide, the out-register arrangement is preferable for Aβ (14–23) dimers. Note as well a critical contribution of aromatic interactions in the dimers stability [19]. Computational studies also suggest that such states are very rare and undetectable in the peptide monomers, but interprotein interactions is the factor that dramatically facilitate the peptide conformational transitions leading to the formation of dimers with extremely high lifetimes, as described above. It is reasonable to assume that similar model is applied to large amyloid proteins. Support for this hypothesis comes from the paper [74] in which structural rearrangements of globular proteins during misfolding were analyzed. The authors proposed that the structural transition to aggregation-prone states occurs at protein-protein contacts formed by the normally assembled proteins.

**Figure 9:**
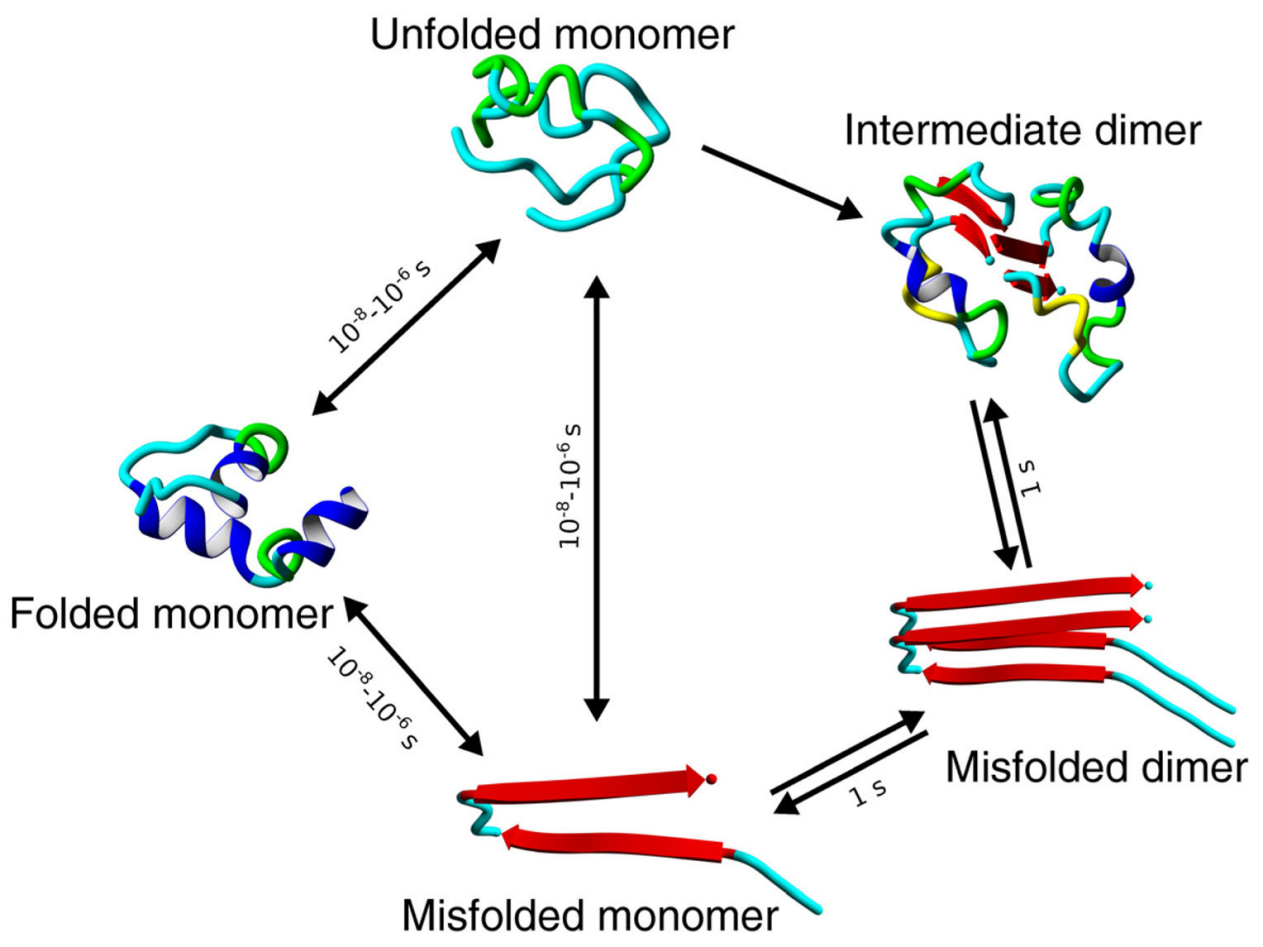
A schematic representation of protein dynamics The transitions of the folded (native) state of the protein to unfolded and misfolded states are shown for simplicity only. Characteristic times for the transition between these states and the dimer lifetime are shown above the arrows.

Combined experimental data and computational analyses lead to a model of protein misfolding and the early stages of the self-assembly process, as schematically explained in Figure 9. The protein monomer can adopt a transient state characterized by unfolding of protein helical segments. For Aβ proteins and its segments, this state will have no helical regions, as demonstrated in Figure 8A for Aβ 14–23 peptide. The timescale of local protein dynamics is in the range of nanoseconds and large-scale dynamics may occur in the range of microseconds [75,76,77,78]. Therefore, we indicate a timescale for protein dynamics for the formation of misfolded conformations in a range of 10 ns–1 μs (10^−6^ s).

According to AFM probing data, when two monomers form a misfolded dimer, the system can live as long as a second. Compared to the dynamics of monomeric proteins, the dynamics of misfolded dimeric conformations are 10^6^ times more stable. This is a very important finding and suggests that dimerization is the mechanism by which a misfolded state of a protein is stabilized. The high stability of dimers indicates that the probability of aggregation occurring in the presence of dimers is 10^6^ times greater than without them. For example, the probability of a tetramer forming by the assembly of two dimers is 10^6^ times greater than the formation of trimers by the interaction of a dimer and a monomer. The difference in the structure of Aβ proteins in monomeric and dimeric forms was reported in [79], in which the dimers were stabilized by crosslinking, and structures of crosslinked oligomers were characterized by CD spectroscopy. The authors also found structural changes between other oligomers, but these changes were minor when compared with the conformational differences between monomers and dimers. Therefore, the ability to form long lifetime dimers is a fundamental property of the misfolded state of a protein, and this dimer formation triggers the protein aggregation process. The described model is a paradigm shift of the protein misfolding and aggregation phenomenon, and highlights the critical role of misfolded dimer formation in this process. Importantly, the proposed model suggests that dimers may be potential targets for the development of treatments for protein misfolding diseases. The key role of the dimer in this process is supported by a number of publications that demonstrate that the most abundant species of Aβ peptides isolated from brain tissue are dimers that have a high neurotoxicity [80]. More recent studies show that naturally secreted Aβ dimers and trimers induce progressive loss of hippocampal synapses [81,82]. The reason for the high stability of dimers isolated from brain tissue remains unclear. It has been proposed that oxidation is one of the factors capable of stabilizing the dimers *in vitro* as well as within the cell [83].

## 6. Conclusions

Studies over the past decade demonstrated that AFM imaging is a valuable tool for the recognition and visualization of objects at the nanoscale level. The capability of AFM to operate at ambient conditions was one of the major factors allowing it to be used for biomedical studies. Moreover, AFM is capable of operating in aqueous solutions to perform topographic studies of fully hydrated samples. The advent of high-speed AFM operating in aqueous solutions combined with video rate data acquisition speed took AFM studies to another level at which biological processes at the nanoscale can be visualized and quantitatively analyzed. This unique capability of high-speed AFM is illustrated by a few examples, but the technique is under development and new applications of high-speed AFM to analyze complex biological systems are on the horizon. In addition to topographic imaging, AFM is capable of measuring intermolecular interactions. Importantly, the same instrument can operate in imaging and force spectroscopy modes, which is a unique feature of AFM. The use of both AFM modalities will advance our understanding of important health related phenomena. The ability of AFM force spectroscopy to quantitatively measure interprotein interactions opens prospects for understanding the molecular mechanisms of the pathogenesis of devastating diseases such as Alzheimer’s, Parkinson’s, and prion diseases. AFM, at the current level of development, is capable of quantitatively characterizing small molecules as potential candidates for the successful treatment of these neurological disorders. The combined use of both AFM modes on the same system, in addition to future technological advances, will aid in the development of treatments targeting different stages of these diseases.
